# Exploring the impact of atmospheric cold plasma technology on plant-based milk analogues and their proteins: A review

**DOI:** 10.1016/j.fochx.2026.103519

**Published:** 2026-01-27

**Authors:** Entesar Hanan, Shivangi Srivastava, Aamir Hussain Dar, Kshirod Kumar Dash, Vinay Kumar Pandey, Rafeeya Shams, Sharath Kumar, Ufaq Fayaz, Ayaz Mukarram Shaikh, Kovács Béla

**Affiliations:** a Department of Food Technology, School of Interdisciplinary Sciences and Technology, Jamia Hamdard, New Delhi, 110062, India; b Department of Food Technology, Harcourt Butler Technical University, Nawabganj, Kanpur, Uttar Pradesh, India; c Department of Food Technology, Islamic University of Science and Technology, Kashmir, India; d Department of Food Engineering and Technology, Ghani Khan Choudhury Institute of Engineering and Technology, Malda, West Bengal, India; e Research & Development Cell, Biotechnology Department, Manav Rachna International Institute of Research and Studies, Faridabad, 121004, Haryana, India; f Department of Food Technology and Nutrition, Lovely Professional University, Phagwara, Punjab, India; g ICAR-Central Institute of Temperate Horticulture, Rangreth, Srinagar, India; h Division of Food Science &Technology, Sher-e-Kashmir University of Agricultural Sciences and Technology Kashmir, India; i Faculty of Agriculture, Food Science and Environmental Management Institute of Food Science, University of Debrecen, Debrecen 4032, Hungary

**Keywords:** Plant milk analogues, Protein structure, Enzyme inactivation, Hydrophilicity, Microbiological deactivation, Protein functionality

## Abstract

Plant-based milk analogues are suitable substitutes for animal milk, more environmentally friendly, and sustainable. The microbiological stability, quality, anti-nutritional elements, and techno-functional properties of plant analogues and their proteins need to be considered. The type and concentration of reactive plasma species are influenced by parameters such as frequency, voltage, electrode spacing, treatment time, and gas composition. These factors regulate the efficacy of cold plasma in microbial disinfection and enzyme inactivation. The results indicate that cold plasma enhances the solubility, emulsifying properties, hydrophilicity, and foaming characteristics of plant-based analogues and proteins. In this study, the most potential sources of cold plasma and their impact on the functional characteristics of plant milk and proteins are thoroughly discussed. The work presents current insights into cold plasma for plant-based milk analogues and highlights their prospective applications, recent research, and developments in the field.

## Highlights

• Cold plasma uses ionized gas to inactivate microorganisms and extend shelf life.
• Cold plasma enhances the solubility and emulsifying properties of milk analogues.
• High voltage and long-duration treatments have detrimental effect on food quality.
• Optimizing cold plasma parameters reduces lipid oxidation and protein aggregation.

## 1 Introduction

The dairy business has prospered due to increasing demand for milk and milk-based products. Milk is a lacteal secretion that comprises a blend of proteins, lipids, carbohydrates, vitamins, and minerals, making it an excellent nutritional resource. Human beings initially domesticated the ruminants to meet the demand for milk and dairy products in the market. Certainly, dairy milk is a highly valuable product that is consumed globally. In 2019, worldwide milk production reached 852 million tons, up 1.4% from the previous year, according to the Food and Agriculture Organisation of the United Nations (FAO). Despite increased accessibility, milk consumption has decreased in recent years (Dharini et al., 2023). Some individuals experience irritation of the intestinal mucosal layer due to sensitivity to components of the mammary gland. The presence of lactose or protein in milk triggers this sensitivity in those who are sensitive to dairy products (Liang et al., 2022). Dairy farming is a significant contributor to greenhouse gas emissions, water consumption, and forest destruction. As a result, this has a direct impact on the United Nations Sustainable Development Goals (Mylan et al., 2019). Dairy product consumption decreased across regions due to numerous factors, including ethical, environmental, and health-related rationales. Many individuals refrain from consuming dairy products owing to health concerns about saturated fats, cholesterol, and lactose.

Plant-based milk analogues denote the non-dairy beverages that are a derivative of plants instead of animals, such as cows, goats, or sheep. These milk alternatives are produced through extracting liquids from diverse plants, seeds, nuts, or grains that emulate the smooth consistency of cow's milk (Aydar et al., 2020). Plant-based beverages often have a smooth texture, making them an apt alternative to milk from milch animals. Although these plant-based analogues are effective substitutes for mammalian milk, the acceptability of these products on the basis of palatability, shelf life, anti-nutritional factors, and allergenicity remains a predicament for their consumption. Numerous investigations are being carried out to address these issues in plant-based milk using various thermal and non-thermal processing methods. (McClements & Grossmann, 2021; Wolf et al., 2020). Conventional heat treatments, viz., pasteurisation, high-temperature short-time (HTST), or ultra-high temperature (UTH), are employed to extend shelf life and maintain microbial protection (Lu et al., 2019). However, this technique may have unfavourable effects on the chemical, physical, sensory, and nutritive qualities (Aydar et al., 2020). In view of these apprehensions, innovative processing techniques that use nonthermal or low-temperature thermal approaches have been widely examined as possible alternatives to conventional heat treatments. These methods can result in efficient deactivation of microorganisms and enzymes without compromising food quality. Thus, in this context, cutting-edge techniques, viz., cold plasma, high-pressure processing, ultra-radiation, microwave treatment, high-intensity ultrasound, ohmic heating, pulsed electric field, and supercritical carbon dioxide, have gained prominence (Aydar et al., 2020).

Cold plasma is a non-thermal processing method that effectively enhances the quality and safety of food products (Dharini et al., 2023). The novel, non-hazardous, operationally adjustable, nonthermal atmospheric cold plasma (ACP) technique is currently attracting considerable attention due to its promising prospects. Plasma, the fourth state of matter, is a non-condensing system that results from ionizing a gas either wholly or incompletely. Cold plasma can inactivate microorganisms through several mechanisms, including the breakdown of cell membranes, damage to DNA, denaturation of proteins, and cell lysis. These combined actions result in the decrease or eradication of microbial populations when applied to plant-based milk analogues. In the case of plant-based milk analogues, instability is also a foremost challenge, which can be addressed through the use of cold plasma techniques that improve the stability and functionality of plant-based milk proteins by enhancing adhesion, hydrophilicity, and resistance to degradation (Ji et al., 2019). Understanding the interaction between plant milk and plasma is vital. Grounded in this research gap, the review aimed to investigate the potential of this groundbreaking technique to enhance the quality and safety of plant-based milk alternatives. The review focuses on how ACP can affect the nutritional profile and protein properties of various non-dairy milks. Furthermore, the review examines the mechanisms by which ACP interacts with plant-based ingredients to inactivate microbes.

## 2 Sources of cold plasma generation and mechanism behind plasma treatment

**Fig. 1:**
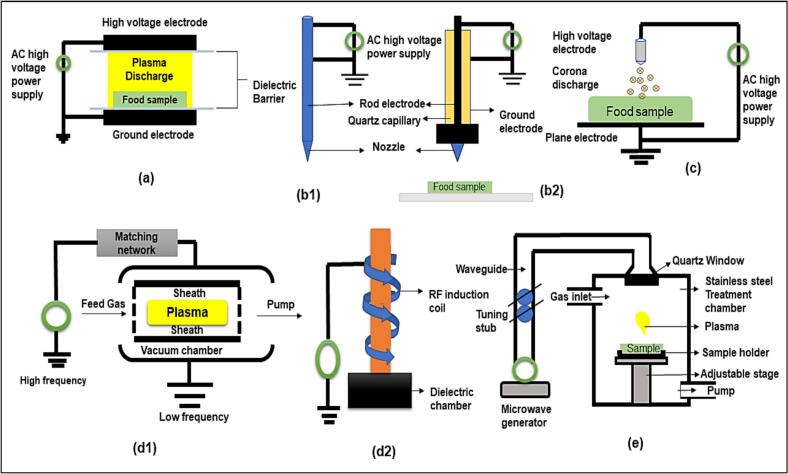
Cold plasma sources: (a) Atmospheric pressure dielectric barrier discharges, (b1) Atmospheric pressure plasma jet, (c) Corona discharges, (d1) Inductively coupled plasma (ICP), and (d2) Capacitively coupled plasmas (CCP), and (e) Microwave plasma source. Non-Dairy or Plant based source of milk.

Ionized gas, called plasma, is considered the fourth state of matter. Electrical neutrality, or the equivalence of positive and negative charges, roughly characterises plasma. According to Coutinho et al. (2018), the plasma state of matter can be achieved by applying thermal, magnetic, or electric energy at radio or microwave frequencies, thereby increasing the kinetic energy of electrons. It is accomplished by partially or fully forming the ionized gas through an electrical discharge. It is mostly composed of ions, atoms, free electrons in their excited or ground states, and photons (Salim et al., 2018). These species are categorised as heavy species (the remaining constituents) and light species (photons and electrons). Thermal and non-thermal, or atmospheric cold plasma (ACP) are the two forms of plasma. A thermal plasma is defined as one in which the temperature of the heavy and light particles is identical. It is created by extremely high heating temperatures (over 9000 °C). ACP is a non-thermal plasma in which the light particles are hotter than the heavier particles, which are at ambient temperature; it is produced at ambient temperatures between 30 and 60 °C (Rathod et al., 2021). A variety of gases, such as nitrogen, argon, helium, heliox (a mixture of oxygen and helium), and air, can be used to create ACP. The various techniques for producing ACP are prescribed in Table 1 and Fig. 1.

**Table 1:** Different techniques of atmospheric cold plasma operation.

Technique	Operation	References
Dielectric barrier discharge (DBD)	This involves utilizing two metal electrodes, connected to a voltage source. The distance between the electrodes can be adjusted, ranging from 0.1 mm to several centimeters (Fig. 1a). The operation of DBD plasma involves a broad spectrum of parameters, including gas pressures within the range of 1 × 10^4^ to 1 × 10^6^ Pa, various frequency bands (ranging from 10 to 50 MHz), and the use of pulsed direct current (DC) or alternating current (AC) with voltage levels ranging from 1 to 100 kVrms.	(Laroque et al., 2022), Feizollahi et al., 2021)
Plasma jet (PJ)	The most distinctive designs are made up of two rings, or coaxial electrodes [Fig. 1(b1 & b2)]. Between the two electrodes, the gas flows. Grounding the outer electrode and applying radiofrequency (13.56 MHz) excitement to the center electrode causes the free electrons to accelerate. This collision with gas molecules produces a variety of reactive species. The gas, flows at a high flow rate (> 10 slm), propels the formed plasma outside the electrode area as a jet and releases the plasma species into the open atmosphere, producing a consistent and uniform discharge at atmospheric pressure.	(Bermudez-Aguirre, 2019). (Nishime et al., 2017)
Corona Discharge (CD)	This type of discharge is exemplified by an uneven pair of electrodes. A point and a plane are molded when the electric field surpasses the collapse edge within a restricted spatial region. When the high electric field close to the electrode is higher than the gas's collapse strength, a weakly ionized plasma is formed (Fig. 1c).	(Scally et al., 2021).
Radiofrequency (RF)	The two main types of RF are ICP and CCP [Fig. 1,(d1 and d2)]. In a CCP setup, two parallel electrodes are positioned a short distance apart within a vacuum chamber. By applying an alternating voltage between these electrodes, (RF) energy is generated. An RF power source, typically operating at 1 kW and at a characteristic frequency of 13.56 MHz, powers these electrodes. This frequency can range from 1 to 500 MHz. The gas is ionized by the RF power source's oscillating electromagnetic field, which forms plasma. The plasma's typical density ranges from 10^15^ to 10^16^ particles per cubic meter when considering both electrons and ions. Typically, ICP systems make use of two RF power supplies. While the primary supply powers a coil that is usually positioned outside the plasma and separated from it by a dielectric window, the secondary supply is in charge of biassing the substrate holder and controlling ion energy (Laroque et al., 2022).	(Bermudez-Aguirre, 2019) (Chizoba Ekezie et al., 2017) (Laroque et al., 2022).
Microwave (MW)	The electromagnetic waves created by a magnetron and used to produce microwave discharges are contained in the MW powered plasma generators; these waves are typically generated at 2.45 GHz (Fig. 1 e). Without the need for electrodes, CP is created when the electrons in gas molecules accelerate due to the microwave electric field. At low and atmospheric pressures, this system can generate plasma. The generated waves are sent through a waveguide into a treatment chamber, where they are absorbed by gas electrons. These electrons interact with microwaves to induce ionization processes through collisions, thereby releasing energy in the form of visible light photons and UV rays.	(Chizoba Ekezie et al., 2017) (Muhammad, Liao, et al., 2018)

Cold (non-thermal) plasma treatment modifies plant milk proteins mainly through the action of **reactive oxygen and nitrogen species (RONS)** such as •OH, O₃, H₂O₂, NO•, and ONOO^−^ together with UV photons, ions, and transient electric fields (Khan et al., 2024). Cold plasma treatment induces changes in the structural and compositional properties of proteins, viz., unfolding, aggregation, and fragmentation, owing to the collective action of free electrons, ions, free radicals, and neutral particles (Bayati et al., 2024). This leads to enhanced emulsification and solubility properties owing to improved surface hydrophobicity and the formation of more flexible protein structures. The plasma components mainly interact specifically at the air–liquid interface and just under the surface of plant milk, promoting controlled chemical and structural changes in the proteins without heating the bulk of the material (Olatunde et al., 2023). At the chemical level, RONS selectively oxidize susceptible amino acid residues, especially Cys, Met, Trp, Tyr and His (Chai et al., 2024). The formation of a disulfide bond as a result of the oxidation of thiols (-SH) alters the arrangement of disulfide bonds (Chen et al., 2025). Similarly, modifications to aromatic residues also alter intramolecular interactions. Under optimal conditions, this oxidative process is usually mild. These chemical modifications cause partial structural unfolding of a range of globular plant proteins. Exposed hydrophobic areas and reactive functional groups caused increased protein–water and protein–protein interactions. However, excessive exposure can lead to irreversible aggregation by cross-linking. Changes in protein conformation induced by plasma at one level result in improvements in protein solubility, emulsifying capacity, and foaming properties at the functional level, as unfolded proteins are more efficiently adsorbed at oil–water and air–water interfaces. Improved surface activity enhances emulsion stability and reduces creaming in plant milk systems. At the interfacial and colloidal levels, plasma affects the surface charge (ζ-potential) of proteins through oxidation and ion interactions (Kumar et al., 2025). They enhance electrostatic repulsion between droplets with a protein coating. Thus, improves dispersion stability. The behavior of protein aggregation is thus shifted toward controlled formation into networks rather than precipitation. Plasma treatment can cause structural damage and oxidative damage to antinutritional factors and enzymes (e.g., trypsin inhibitors, lipoxygenase-linked proteins), rendering them inactive from a bioactivity and safety perspective.

## 3 Non-dairy or plant-based milk analogues and proteins

Throughout the ages, milk has been an important component of the human diet. Milk derived from mammals is considered a nutritious food due to its vital constituents, including proteins, minerals, vitamins, lipids, and lactose (Haas et al., 2019). However, the primary issues revolving around mammalian milk include milk protein allergies and lactose intolerance, as well as hormone levels and the use of antibiotics in cattle. In light of the aforementioned information, consumer demand for non-dairy beverages and alternatives to cow's milk has increased significantly, reaching 61% since 2012. The demand for non-dairy milk has been driven by various factors, including the adoption of modern dietary practices, vegetarian and vegan lifestyles, environmental considerations, and ethical objections to the use of cow's milk (Reyes-Jurado et al., 2023). The proliferation of innovative plant-based milk analogues in large retail stores indicates the high customer demand for these products (Bocker & Silva, 2022; Walsh & Gunn, 2020). The effect of cold plasma treatment on different properties of plant-based milk analogues and proteins is presented in Table 2.

**Table 2:** Effect of cold plasma treatment on different properties of plant-based milk analogues and proteins.

Plant Milk/protein	Processing conditions	Parameters Studied and their outcome	References
Coconut milk	ACP (Discharge voltage 50,60 and 70 kV, treatment time 30.60.90 s)	**Viscosity-** Viscosity reduced with the increase in shear rate **Emulsification-**Enhanced **Particle Size-** Decreased **pH-**Decreased **TA-** Increased **TSS**- Increased **Protein-** Non – significant change **Fat-** Non – significant change **Colour-** No significant change in L*, a* increased, b* decreased, ΔE increased **Fatty acid-** MCFA increased **POV-** Insignificant difference **TBARS**- Increased **A**min**o Acid-** amino acid contents decreased	(Chen et al., 2024)
Sesame milk	Cold plasma bubbling (constant power level 180 V, constant flowrate 1 l/h and treatment time 10,20,30 min.	**Viscosity**- Viscosity reduced on plasma treatment **Microbial load-** Decreased **pH-**Decreased **TA-** Increased **Colour-** L* decreased, a* and b* increased, ΔE slightly changed. **TBARS**- Increased **Amino Acid-** amino acid contents increased	(Dharini et al., 2023)
Soy milk	Plasma bubbling (100 and 200 V voltage levels for 5, 15, and 25 min)	**Viscosity**- Viscosity increased on plasma treatment **pH-**Increased Protein- Non – significant change **Colour-** WI Decreased **FTIR-** β sheets increased **Antigenicity ELISA-** Decreased **SDS PAGE-**No protein band was added	(Anbarasan et al., 2023)
Soy protein isolate (SPI)	ACP (voltages, 170,200, 230 V, exposure time 5, 10, 15 min	**Heat Stability-** Decreased **FTIR-** β sheets increased **SDS PAGE-**Decrease in molecular weight bands	(Dabade et al., 2023)
Oat milk	Plasma treatment (pin to plate plasma reactor); (voltage 170,200,230 V, time 5,10,15 s)	**Viscosity**- Viscosity reduced with the increase in shear rate **Particle Size-** Decreased **Microbial load-** Decreased **pH-**Decreased **TA-** Increased **TSS**- Increased **Protein-** Decreased **Colour-** No significant change in L*, a*, b* and ΔE	(Eazhumalai et al., 2022)
Soyabean protein isolate (SPI)	ACP (frequencies (80 to 120 Hz) and durations (1 to 10 min)	**Solubility:** Increased, while it suffered a reduction within 5 min followed by an increase at 10 min. **Emulsification-**Enhanced **Particle Size-** Decreased **Amino Acid**- SH group were gradually decreased **Antigenicity ELISA-** Decreased **SDS PAGE-** No noticeable changes in the molecular weight bands	(Zhang et al., 2021)
Flaxseed protein	ACP (Voltage 5 kV, frequency 40 kHz and treatment time 0–240 s)	**Solubility:** Decreased. **Emulsification-**Enhanced **Particle Size-** Increased **pH-**Decreased	(X. Yu, Huang, et al., 2020)
Peanut protein lactose conjugate	DBD (90 W, treatment time 0 to 5 min)	**Solubility**: Increased up to 3 min and then decreased **Heat Stability-** Increased	(J. jiao Yu, Huang, et al., 2020)
Peanut protein isolate dextran conjugate	DBD (voltage 35 V, treatment time 0.5 to 3 min	**Heat Stability-** Increased **FTIR-**β sheets increased, β-turns decreased **Amino Acid-** amino acid contents decreased **SDS PAGE-** Creation of high and low molecular weight bands at top and bottom	(Ji et al., 2020)
Peanut	ACP (Frequency 52 kHz, voltage 32 kV)	**Antigenicity ELISA-** Decreased **SDS PAGE-** No changes up to 15 min followed by slight change on increase in treatment time	(Venkataratnam et al., 2020)
Tiger nut milk	DBD plasma treatment (30 V and resonance 1.22 A, exposure time 0–12 min).	**Microbial load-** Decreased **pH-**Decreased **TA-** Increased **TSS**- Increased **Protein-** Decreased **Fat-** Non – significant change **TBARS**- Increased	(Muhammad et al., 2019)
Peanut protein isolate	ACP (Voltage 70 V, treatment time 1 to 10 min	**FTIR-** β-turn increased, β-sheets decreased	(Ji et al., 2019)
Peanut protein	DBD (voltage 35 V, treatment time 1,2,3 and 4 min)	**Solubility:** Increased up to 3 min and then decrease **Heat Stability-** Decreased **Particle Size-** Increased **pH-**Mild Decrease **FTIR-**β-sheets and random coil increased, of α-helix and β-turn decreased **Amino Acid**- Progressive decrease in SH group **SDS PAGE-**No change in electrophoresis profiles	(Ji et al., 2018)
Soy protein isolate	ACP	**Antigenicity ELISA-** Decreased **SDS PAGE-**Disappearance of protein bands	(Meinlschmidt et al., 2016)

**Fig. 2:**
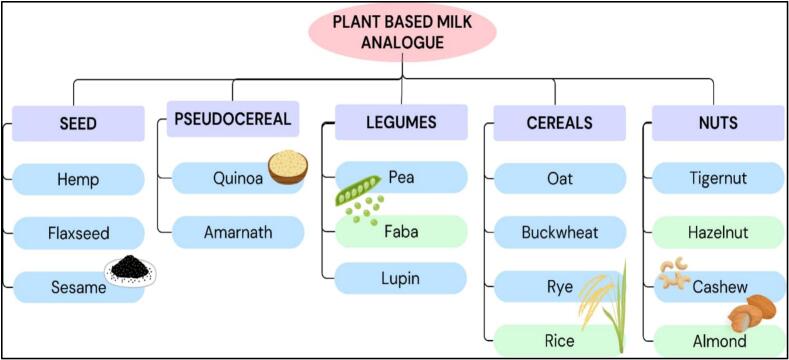
Sources of Plant-based milk substitutes.

Plant-based milk substitutes are beverages derived from plant elements, such as nuts, cereals, oilseeds, legumes, and pseudo-cereals (Fig. 2). Following water extraction, products are homogenised to achieve a particle size distribution of 5–20 μm, resembling the texture and appearance of dairy milk (Bocker & Silva, 2022). Non-dairy alternatives are beneficial for health and can help lower the risk of diseases such as diabetes, cancer, atherosclerosis, and cardiovascular disease. This is due to the presence of fibre, isoflavonoids, antioxidants, and fatty acids (Aydar et al., 2020). However, they have several disadvantages, such as low protein content, limited absorption of vitamins and minerals, and reduced levels of essential micronutrients and amino acids, such as lysine, cysteine, and methionine, which are vital for maintaining normal body functions. The incorporation of vitamins and minerals into plant-based beverages can effectively fulfil their nutritional requirements. Moreover, a blend of plant matrices can be used to manufacture the products, thereby increasing the amount of amino acids. The outline of the diverse types of existing plant-based milk equivalents, their proteins, and their production processes at a laboratory level using cold plasma, is illustrated below.

### 3.1 Coconut milk

Coconut milk is mostly used in Southeast Asian cuisine. It is consumed as a drink and also used as a component in many savoury and sweet cuisines. The processing of coconut milk starts with finely grating the coconut and then soaking the grated coconut in hot water. The resulting mixture is then strained to obtain the milk extract. Coconut milk is usually used as a thickening agent, as it is a good source of fat. However, the protein content of the coconut milk is lower. Coconut milk is reported to have numerous health benefits, as well as anti-viral, anti-microbial, anti-carcinogenic, and anti-bacterial properties (Sethi et al., 2016). It is reported to be high in lauric acid, which supports immune system function, stimulates brain development, and upholds blood vessel flexibility. It is also a good source of caprylic and capric acids. Additionally, it contains many antioxidants, such as vitamin E, which helps prevent ageing (Abdullah et al., 2018). Furthermore, there are no major allergens present, and the availability of iron, magnesium, and copper ions is high. Coconut milk has a rich, creamy texture and a milky tint.

**Fig. 3:**
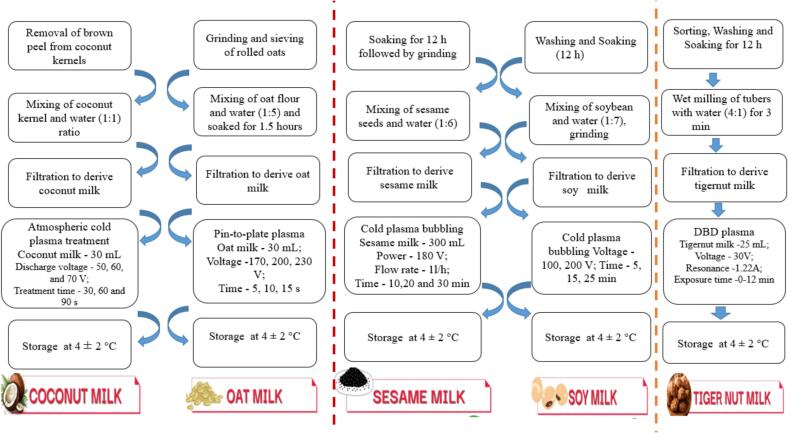
Flow chart for plasma processing of coconut milk, oat milk, sesame milk, soy milk, and tiger nut milk.

The manufacture of coconut milk using cold plasma processing was documented by Chen et al. (2024), as shown in Fig. 3. The coconut kernels were mixed after removing the brown peel from the coconuts. Coconut milk was extracted by combining equal parts of coconut kernel and water (1:1). After that, the 80 ml of coconut milk was placed within a 180 mm by 150 mm by 50 mm polypropylene food tray, covered with packaging film, and processed using an ACP machine. Two polypropylene sheets are jammed between two aluminium electrodes in the ACP system, with a polypropylene food tray in the centre. ACP was used to treat coconut milk with a 40 mm gap and a 10 mm discharge gap. The treatment duration was set to 30 s, 60 s, and 90 s in air, and the discharge voltage was set to 50 kV, 60 kV, and 70 kV. Following the treatment, the treated coconut milk was then stored at 4 ± 2 °C for 12 h.

### 3.2 Oat milk

Presoaked oat groats are used to make oat milk. Usually, they are cracked pieces of hulled grain. Because oat milk is lighter than other kinds of milk, it works well in light cream soups and curries. It has a somewhat sweeter texture, similar to fat-free or low-fat cow's milk, making it suitable for baked goods. It can be used in savoury and sweet recipes. It is high in β-glucan fibre, which lowers total and LDL cholesterol, helping lower blood glucose and promoting hypocholesterolemia. It is also found to have significantly higher fat and carbohydrate content than the other cereals. Oats are gluten-free and have no significant allergens (Basinskiene & Cizeikiene, 2019). One of the drawbacks is the high starch content, which makes emulsification difficult; antinutrients (phytates and trypsin inhibitors); low calcium content; and high lipase levels, which can cause it to go rancid. In oats, lysine is a limiting amino acid (Vanga & Raghavan, 2018). The composition of oats consists of 60% carbohydrate, 5–9% lipids, 11–15% total protein, 0.54% calcium, and 2.3–8.5% dietary fibre. This high starch concentration makes it difficult to form a stable emulsion, and when oat beverages are heated, the starch starts to gelatinise, making the liquid milk more viscous and gel-like, which lowers its acceptability. Therefore, alpha- and beta-amylase are used in the enzymatic hydrolysis process to overcome this and achieve a suitable result.

The cold plasma processing method for preparing oat milk is shown in Fig. 3 (Eazhumalai et al., 2022). The rolled oats were ground into fine particles in an electric grinder and sieved through a 0.850-mm mesh to create oat milk. Weighed and soaked the sieved oat flour in water at a 1:5 (*w*/w) ratio for 1.5 h; the mixture was then stored in the refrigerator at 4 ± 2 °C. After thoroughly mixing the slurry, it was filtered through muslin fabric. After the oat milk was collected, it was kept at 4 ± 2 °C in an airtight container. Next, the oat milk samples were treated with cold plasma in a unique pin-to-plate plasma reactor. The plasma generator configuration consists of two stainless-steel plate electrodes: the upper electrode, which is the high-voltage electrode with 88 pins spaced 5 cm apart from the bottom plate, is the ground electrode. The duty cycle, discharge frequency, and resonance frequency were all kept at 1100 Hz, 90 μS, and 55.51 Hz, respectively, as the other treatment parameters. A 30-ml sample of oat milk was placed in a 12-cm-by-12-cm polyethene terephthalate (PET) plate with a 0.3 mm depth. Three distinct exposure durations (5, 10, and 15 min) and three distinct input voltage levels (170, 200, and 230 V) were used for the treatment. After processing, the samples were placed in sterile, airtight Falcon tubes and stored in a refrigerator at 4 ± 2 °C.

### 3.3 Sesame milk

One of the major oilseed crops grown worldwide is sesame. It is a premium protein source with a unique sulfur-containing amino acid balance. A considerable number of lignans, which have been shown to have nutraceutical properties such as antioxidative, hypocholesterolemic, anticarcinogenic, anticancer, and antiviral activity, are found in sesame seeds. According to Vanga and Raghavan (2018), it has an excellent lipid profile and is low in saturated fatty acids. The primary fatty acids are oleic (18:1), linoleic (18:2), palmitic (16:0), and stearic (18:0). A significant amount of phytates and oxalates, two anti-nutritional agents, are present in sesame. However, oxalates are only found on the hull's surface, and most are removed during decortication, which also improves flavour. One limiting amino acid is lysine. Sesame proteins are less soluble in water and more prone to heat denaturation, which limits their utilization and is one of the primary obstacles in the manufacturing of sesame milk. Therefore, before using sesame protein in the manufacture of milk, its functionality must be altered. Since the functional characteristics of sesame proteins can be altered by a variety of processing techniques, including soaking, defatting, roasting, fermentation, germination, and microwave heating, these techniques have been reported (Silva et al., 2020). The adverse effects associated with soy milk consumption, viz., allergies, flatulence, and off-flavour, are typically uncommon with sesame milk consumption. The nutty, creamy flavour of sesame milk makes it ideal for developing milk substitutes. Researchers have found that when it comes to calcium, protein, unsaturated fatty acids, and fats, sesame substitutes outperform soy-based beverages in terms of nutrition (Dharini et al., 2023).

The production of sesame milk by means of cold plasma processing was studied by Dharini et al. (2023), as shown in Fig. 3. In summary, newly harvested whole sesame seeds were cleaned, submerged in distilled water for a whole day, and then ground in a household blender. The ratio used was 1 part sesame seeds to 6 parts water. The mixture was strained and filtered through two layers of muslin linen to extract the sesame meal. The filtered sesame extract/milk was used exactly as is for the plasma treatment. A Dielectric Barrier Discharge (DBD) unit, which consists of an outer hollow aluminium cylinder that acts as an electrode and an inner meshed electrode that acts as the ground electrode, is the basis for the Plasma Bubbling (PB) unit that was part of the processing apparatus. Between these electrodes was a hollow quartz tube that served as a dielectric barrier inside the DBD unit. The DBD unit was linked to a blower unit, which allowed ambient air to circulate between the quartz tube and electrode. The hollow tube had openings on both sides for the air intake and output. The energized electrodes ionize the gas molecule. This PB unit was used to treat the samples of sesame milk. The sesame milk sample received bubbles containing ionized air from the PB unit. A constant power level (180 V) and constant flow rate (1 l/h) were used to bubble 300 ml of milk, with variations in the bubbling time (10, 20, and 30 min).

### 3.4 Soy milk

Because soy milk has the highest protein content, it is similar to cow's milk. The earliest reports of soy milk date back roughly 2000 years in China. It was the first plant-based milk analogue, designed to provide nutrition to people in areas where milk wasn't readily available. Filtered water and ground soybeans are used in its production. It might include tastes and sweeteners. It has a silky, creamy texture. It has a nutty, slightly sweet flavour. One of the best kinds of milk to use in cooking is soy milk. It's the perfect option for savoury recipes and sauces thanks to its strong temperature stability. This makes it an excellent option for baking because of its high protein content. Isoflavones (Genistein), phytosterols, and important mono- and polyunsaturated fatty acids found in soy milk are beneficial for cardiovascular health and have anti-cancer and anti-osteoporosis properties. The output of the conventional soy milk preparation method has a distinct beany flavour and a short shelf life. Modern soy milk manufacturing combines state-of-the-art methods and equipment to minimise beany flavour and maximise nutritional content, shelf life, and convenience (Sethi et al., 2016). Astolfi et al. (2020) report that soy milk contains high levels of magnesium, iron, and copper ions, as well as high levels of PDCAAS and DIAAS. Its proteins' amphipathic nature explains its acceptable emulsifying properties. Three phytochemicals have been identified: phytic acid, saponins, and sterols. According to Chalupa-Krebzdak et al. (2018), there is also a considerable amount of polyunsaturated fatty acids, especially linoleic (18:2) and linolenic (18:3) acids. The presence of potentially allergenic proteins, the high levels of antinutrients (trypsin inhibitors), and the off-flavours (astringency and beany aromas) are the main negatives. Moreover, methionine and cysteine are limiting amino acids. The soy milk production via cold plasma application was demonstrated by Anbarasan et al. (2023), as depicted in Fig. 3. Briefly, soybean seeds were initially soaked in water for 12 h. After draining the water, the soaked soybeans were mixed with distilled water at a 1:7 ratio and then ground. The resulting mixture was filtered using a double-layered muslin cloth. The obtained soy milk was subsequently treated with plasma bubbling, varying the voltage (100 V, 200 V) and duration (5, 15,25 min). A blower pump was used to maintain a constant input voltage of 80 V and regulate airflow to 1 L/min. Following the treatment, all samples subjected to plasma bubbling were collected.

### 3.5 Tiger nut Milk

In Africa, Asia, and a small portion of Europe, tiger nuts are grown. Tiger nut is documented to be a rich source of fat and carbohydrates, and an outstanding mine of potassium, phosphate, and vitamins E and C. The sweet-tasting tiger nut milk (TNM) with low acidity is derived from a tuber. The composition of the tuber depends on the region where it is grown. The use of TNM has been linked to averting thrombosis, colon cancer, and heart attacks. It has an impressive biological value. It has a moderate fat content, with substantial quantities of linoleic (18:2) and oleic (18:1) acids. Carbohydrate content is high, ranging from 12 to 17%. According to Codina-Torrella et al. (2017), it has a low glycemic index. Other quality criteria that are linked to TNM quality loss include starch and protein content, pH, enzyme activity, lipid oxidation, and TNM shelf life. Typically, the protein content is less than 1%, which is the primary constraint.

Muhammad et al. (2019) detailed the preparation of TNM (Tiger Nut Milk) using cold plasma processing, as illustrated in Fig. 3. Initially, tiger nuts were separated to remove any broken tubers. Approximately 200 g of cleaned tiger nuts were soaked in deionised water at room temperature for 12 h. After draining the water, the succulent tubers were mixed with deionised water (4:1, *w*/w) and wet-milled for 3 min. The resulting milky extract was then filtered via a muslin cloth to obtain TNM. This TNM was then treated with DBD (Dielectric Barrier Discharge) plasma, utilizing ambient air as the process gas. The plasma apparatus included a voltage regulator, a glass dielectric barrier with a 90 mm diameter, a reaction cell with 50 mm diameter aluminium electrodes, and a high-frequency power supply. The resonance balance was set to 1.22 A at 30 V for this setup, with the powered electrode positioned 6 mm away from the sample surface. A 150 mm stainless steel dish containing 25 ml of TNM provided a sample depth of 0.5 mm for the plasma treatment. DBD plasma was then applied to the TNM while continuously stirring for exposure durations of 0, 2, 4, 6, 8, and 12 min. The TNM was rotated in the dish using a glass rod to agitate it without disrupting the plasma discharge for the predetermined treatment duration.

### 3.6 Soy protein

Soy protein is an extensively consumed source of protein. It has numerous uses in food processing and product development, as it is a pure protein (90%). The main proteins in soy are β-conglycinin (7S) and glycinin (11S). It also contains some polar and non-polar amino acids. The protein's properties, viz., its interactions with water and enzymes, are determined by its charged structure. The stable structure of glycinin and its molecular flexibility are due to the disulphide bond and electrostatic and hydrophobic interactions, respectively. Therefore, the properties of soy protein can be altered using various techniques, such as atmospheric cold plasma (Sharafodin & Soltanizadeh, 2022).

**Fig. 4:**
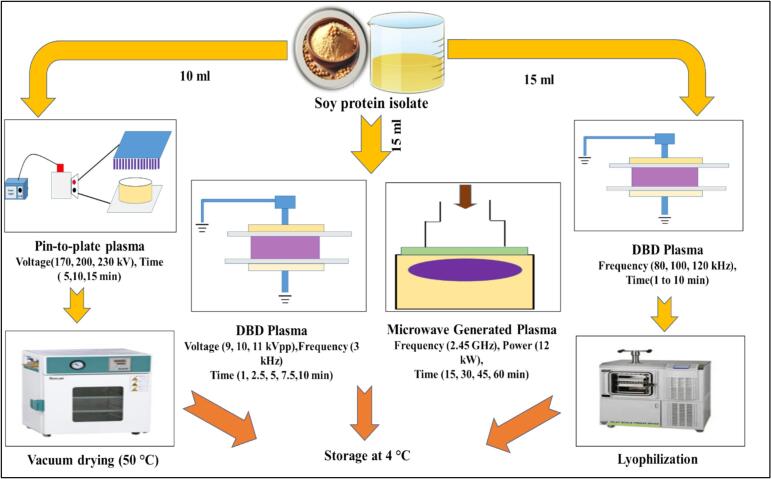
Flow chart of soy protein plasma processing.

Dabade et al. (2023) conducted an ACP treatment on Soy protein isolate (SPI) using a pin-to-plate cold plasma reactor. The liquid SPI solution (10 ml) was placed on the petri plate on the sample stage, sandwiched between the 2 metal electrodes. Different ACP treatments were given at different input voltages (170, 200 and 230 V) and time intervals (5, 10, and 15 min). To regulate time and voltage, a step-up transformer was used. The treated sample was then dried in vacuum at 50 °C and stored for further use. In another study, Zhang et al. (2021) used DBD plasma to treat SPI. Briefly, SPI solution (15 ml) was placed in a petri plate and packed into a polyethene terephthalate tray (178 × 126 × 35 mm), which served as a close reactor and an additional dielectric barrier. The distance between the 2 electrodes was kept at 35 mm. The SPI sample was treated with cold plasma at 40 kV, 80, 100, and 120 Hz, and for 1 to 10 min. After the treatment, the sample was lyophilized and stored. Meinlschmidt et al. (2016) reported the use of two plasma treatments for SPI, viz., direct plasma treatment using a surface DBD system and indirect plasma treatment using a microwave-generated plasma. In the case of surface DBD, SPI (15 ml) was placed in a petri dish on a holder and subjected to the plasma source at a distance of 12 mm. During the plasma treatment, the airtight chamber was filled with ambient air and sealed hermetically. Plasma generation was performed using a commercial arbitrary waveform generator by applying sinusoidal peak-to-peak (pp) voltages of 9, 10, and 11 kVpp at a frequency of 3.0 kHz at the surface of the dielectric epoxy glass. A controlled voltage was applied via an in-built oscilloscope, and the exposure time was set to 1, 2.5, 5, 7.5, and 10 min without stirring. The samples were then stored at −20 °C. In the case of indirect plasma treatment, the SPI (15 ml) was placed in a reaction chamber, which was then filled with plasma-processed air (PPA). The PPA was generated by a microwave-driven plasma torch operating at 2.45 GHz and 12 kW, using air as the process gas, with a gas flow of 18 slm, and a plasma peak temperature of 3700 K. For a standardised treatment, the bottles were shaken. The treatment times were 15, 30, 60, and 90 min, and the temperature was kept below 30 °C. The treated samples were then kept at −20 °C. The different plasma-processing methods for soy proteins are illustrated in Fig. 4.

### 3.7 Peanut protein

Peanuts are a significant oilseed crop. Peanuts are primarily used for oil extraction. The byproduct, Defatted peanut flour (DPF), is a rich source of protein, containing about 47–55% high-quality protein. The DFP is widely used as a cholesterol-free substitute for commercial animal protein. Peanut protein mainly comprises arachin (14S), arachin I (7.8S), and arachin II (2S). Arachin (14S) is the major protein fraction in peanut protein and accounts for ∼75% of the total peanut protein. However, the functional properties, viz., emulsifying, foaming, and gel-forming, of peanut proteins are poor, which constrain their use. Keeping this in view, ACP is being explored as part of the array of protein modifications (Ji et al., 2019).

**Fig. 5:**
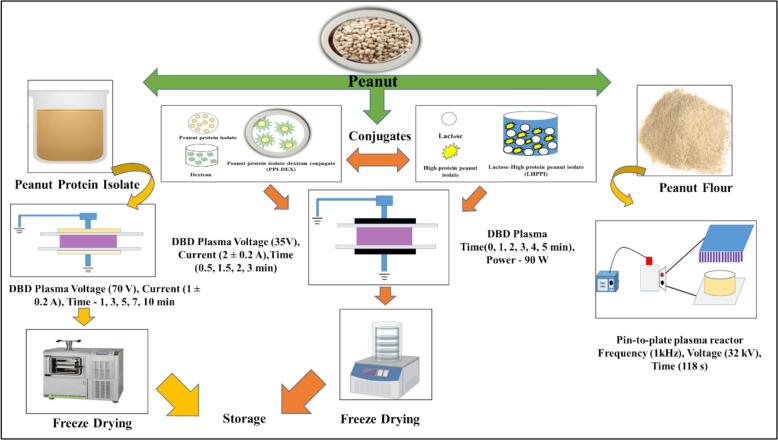
Flow chart of peanut protein plasma processing.

Ji et al. (2019) reported the use of DBD for the treatment of PPI. For treatment, the input voltage, discharge current, and discharge time were 70 V, 1 ± 0.2 A, and 1, 3, 5, 7, and 10 min, respectively, and the treatment conditions were 50 ± 1% RH and 25 ± 2 °C. In another study, Ji et al. (2018) treated PPI (10 ml) with DBD plasma. The PPI solution was applied to the bottom dielectric barrier and plasma-treated at 35 V and 2 ± 0.2 A for 1, 2, 3, and 4 min, respectively. The treatment conditions were 45 ± 1% RH and 20 ± 2 °C. The treated samples were then freeze-dried. Several other researchers also reported the use of DBD plasma processing for PPI conjugates. Ji et al. (2020) in his study treated the Peanut protein isolate- dextran (PPI-Dex) conjugate (10 ml) with DBD plasma. The treatment parameters and conditions were, voltage 35 V, current 2 ± 0.2 A, treatment time 0.5, 1.5, 2 and 3 min, and RH 45 ± 1%, temperature 25 ± 2 °C respectively. The treated samples were then freeze-dried and the powdered samples were then stored till further use. Likewise, Yu, Huang, et al. (2020) in his findings reported using DBD for treating Lactose-High protein peanut isolate (L-HPPI). The process parameters used were discharge power of 90 W for 0, 1, 2, 3, 4, and 5 min. Finally, the L-HPPI conjugate powders were obtained via freeze-drying and then stored at −20 °C until further use. In another study, Venkataratnam et al. (2020) reported the use of a novel large-volume pin-to-plate atmospheric plasma reactor to reduce the allergenicity of the major peanut allergens Ara h 1 and Ara h 2. Briefly, the defatted peanut flour (DPF; 10 g) was placed in a petri dish, which was in turn placed between the two steel plates acting as high-voltage electrodes, comprising a pin array (11 × 8) and a flat plate ground electrode. The electrode pins help produce a small convex shape that encourages a consistent discharge of plasma across the array, with the core pins closer to the ground electrode. A 7 cm distance was kept between the bottom of the pins. The experimental conditions for generating a plasma discharge were a resonant frequency of 52 kHz, a discharge voltage of 32 kV, a discharge frequency of 1 kHz, and a duty cycle of 118 s. Plasma processing for peanut protein isolate, conjugates, and flour is depicted in Fig. 5.

### 3.8 Flaxseed protein

Flaxseed protein is said to have several health benefits. It contains the high-molecular-weight (11-12S globulin) and low-molecular-weight (1.6-2S albumin) salt-soluble fraction and the water-soluble fraction, respectively. Flaxseed is reported to contain 34.3% essential amino acids, making it a potential source of protein. The techno-functional properties of flaxseed protein, viz., gel, foaming, and emulsifying properties, are attributed to the presence of apolar amino acids. The techno-functional properties are also partially attributed to the polysaccharide mucilage migrating during the extraction process. Hence, the researchers are exploring how ACP modulates the techno-functional properties (S. Wu et al., 2019). Yu, Huang, et al. (2020) in their study treated a flaxseed protein solution (FPS) (150 ml) with an atmospheric-pressure plasma jet (APPJ) based on a gliding arc discharge in air over the solution surface. A high-voltage, high-frequency generator (5 kV, 40 kHz) was used to apply the voltage and frequency. The APPJ nozzle had a 5.0 mm diameter and a discharge power of 750 W. A 30 mm gap was maintained between the nozzle and the solution surface. The working gas was compressed air, with a flow rate of 30 L/min at the jet outlet. APPJ treated the FPS for 0, 5, 10, 15, 30, 60, 90, 120, and 240 s. The sample solution vials were soaked in an ice bath to maintain the temperature below 40 °C.

### 3.9 Impact of cold plasma on functional, structural, and compositional solubility

Enhancing protein solubility can be aided by cold plasma, which alters protein structure. It is usually accomplished by protein aggregate breakdown and the addition of specific hydrophilic groups, as a result of reactive species produced during cold plasma treatment, leading to conformational changes. Better contact and interactions between water molecules and the protein surface are made possible by these newly added groups. However, extensive interactions with water during prolonged exposure can result in overcrowding of protein micelles. After a given amount of exposure, overcrowding reduces the number of available active sites for reactions, eventually leading to a drop in solubility. It has been reported that the short-term plasma treatment increases protein solubility due to the uniform particle size (Mahdavian Mehr & Koocheki, 2020). Moreover, it has been demonstrated that prolonged treatments can lead to intra- and intermolecular cross-linking, resulting in supramolecules with larger hydrodynamic radii. Thus, it is essential to regulate process parameters to avoid cross-linking events that promote aggregation and reduce solubility when the protein's maximum solubility is required (Basak & Annapure, 2022). Zhang et al. (2021) found that adding CP to SPI dispersions improved protein solubility compared to the untreated sample. A 282% increase in the solubility of SPI treated for 3 min at 120 Hz was observed. X. Yu, Huang, et al. (2020) found that the relative protein solubility of the flaxseed protein dispersion changed slightly from 0 to 15 s after APPJ treatment. As exposure time increased, relative protein solubility decreased linearly, reaching a minimum of 56.90 ± 3.61% after 240 s of APPJ treatment. The spatial conformation breakdown of flaxseed protein revealed previously hidden hydrophobic regions within the protein molecules. The main factor in the aggregation and gradual loss of solubility of flaxseed protein was the interaction of its hydrophobic groups. Higher levels of peanut protein are probably responsible for the initial increase and subsequent reduction in solubility, as reported by Ji et al. (2018). As previously reported by Yu et al. (2021), a similar pattern was observed for the solubility of the lactose conjugate and high-temperature peanut protein (L-HPPI) following cold plasma treatment. Protein solubility and activity are increased by the Maillard reaction, which also promotes the formation of covalent bonds between proteins and polysaccharides or smaller reducing sugars, thereby inhibiting aggregation. The conjugates' solubility rose from 0.61 mg/ml to 1.01 mg/ml after one minute of isolation heating. Moreover, this solubility increased with increasing cold plasma treatment time, reaching 1.34 mg/ml after 3 min. However, with extended plasma treatment, a decline in solubility was observed as sugar molecules occupied all the protein's active sites. The decline was also attributed to a decrease in the number of hydrophilic groups on the protein surface and to protein aggregation.

### 3.10 Emulsification

The ability of plant proteins to emulsify is their primary characteristic and is intimately linked to increased protein solubility. For protein to have an emulsifying effect, it must rapidly adsorb at the water-air or water-oil interface, intermingle with adjacent molecules, and form a strong cohesive film (Sharafodin & Soltanizadeh, 2022). By altering the protein confirmation, cold plasma can significantly affect the protein structure in plant-based milk, leading to enhanced emulsification properties owing to improved surface hydrophobicity and the creation of more flexible protein structures that allow better oil-water interactions. However, excessive treatment can result in protein accretion and adversely affect emulsification. Chen et al. (2023) in a study on coconut milk stated that by varying the atmospheric cold plasma (ACP) treatment conditions, the protein structure may change, which then directly influences the emulsifying characteristics of the proteins. By means of inadequate ACP treatment, only a marginal change in the protein structure of coconut milk was reported, thereby revealing comparable droplet sizes in the treated and untreated coconut milk. The moderate ACP treatment, however, increased the size of the protein structure and unveiled hydrophobic groups, thereby facilitating protein-water binding at the oil-water boundary and improving the protein's emulsifying capacity. However, severe ACP therapy led to protein aggregation, thereby diminishing the emulsifying ability of coconut milk. Large droplets were significantly reduced in size by the ACP treatment, especially when applied at 60 kV for 60 s, thereby increasing the protein's emulsifying activity. Low-frequency short-term plasma can lead to more flexible structures and improve emulsifying properties. Zhang et al. (2021) provided a clear illustration of this, demonstrating that soy protein isolate treated at a low frequency (80 Hz) had better emulsifying properties than at a higher frequency (120 Hz). High frequencies can cause protein breakdown by forming insoluble protein clumps, which negatively affect the protein's interfacial characteristics. While emulsifying activity increased at lower frequencies and decreased at higher frequencies, stability showed the opposite trend. One possible effect of CP's partial unfolding and elimination of protein clumps is an enhanced emulsification capacity. One of the primary dominant mechanisms of flaxseed protein is its unfolding, which has been shown to enhance its emulsifying activity (X. Yu, Huang, et al., 2020). The oxidative changes in amino acid side-chains, the interactions between proteins and flaxseed-free phenolic acids, and the depolymerisation products of lignan oligomers all affected emulsifying activity. The APPJ treatment for 5 to 10 s significantly improved the stability and emulsifying capacity of flaxseed protein. However, these qualities steadily decreased as the duration of the APPJ treatment increased (15–240 s). With an increase in the APPJ treatment time, the insoluble flaxseed protein particles and the trace amounts of flaxseed protein and polysaccharide complex coacervation brought on by the pH value decrease may gradually absorb the oil-water interface of emulsions, affecting the emulsifying properties of flaxseed protein.

### 3.11 Viscosity

It is often observed that treatment with cold plasma reduces the viscosity of plant-based milk analogues. This decrease can be explained by the interaction of protein and starch molecules with highly energized plasma components, such as excited molecules, reactive species, atoms, and free radicals. As a result, especially at greater concentrations of free radicals, this interaction weakens the fragile structure of these polymeric chains and breaks hydrogen bonds. Moreover, this would result in lower flow resistance, thereby lowering viscosity (Wang et al., 2018). Several studies have reported a reduction in viscosity after CP therapy. The viscosity of coconut milk was examined by Chen et al. (2024) at a range of shear speeds (1–100 s^−1^) and ACP treatment parameters. It was found that the viscosity was high at low shear rates and gradually decreased to a constant value as the shear rate increased. The results showed that coconut milk displayed a shear thinning behavior characteristic of non-Newtonian fluids because an increase in shear rate was destroying the structure of the emulsion. Moreover, variations in coconut milk viscosity were observed across different ACP treatment conditions. When too much or too little ACP was added, the viscosity was comparable to raw coconut milk. Similarly, Dharini et al. (2023) examined the effect of CP on the viscosity of sesame milk. They demonstrated that plasma-treated samples had lower viscosity, likely due to modifications in the protein's structure or a decrease in the size of suspended particles in sesame milk. For oat milk, Eazhumalai et al. (2022) found comparable outcomes. A non-Newtonian pseudoplastic flow characteristic was observed as the shear rate increased, leading to a decrease in viscosity. It was discovered that the lipid, protein, and starch compositions of the oat milk were altered, causing the viscosity to decrease after exposure to plasma. Additionally, aggregation in oat milk due to the presence of proteins and carbohydrates also results in decreased viscosity. Significantly, viscosity was reduced by plasma treatment at higher voltages (200 and 230 V) and longer exposure times (15 min), with a minimum of 20 cP at the highest shear rate. In contrast, at a voltage of 170 V, the lowest viscosity—roughly 40 cP—was recorded. There was a discernible difference between the samples treated with plasma and the control group. Anbarasan et al. (2023), in their study on soy milk, however, reported contrary results and stated that viscosity increased when soy milk was subjected to plasma bubbling (PB). An increase in the viscosity from the initial value (4.13 ± 0.30 cP) to the final value (4.90 ± 0.15 cP) at 15 min and (4.90 ± 0.39 cP) at 25 min at 100 and 200 V, respectively, was noted. This increase in viscosity is attributed to the formation of foam on the soymilk surface as a result of PB treatment, which eventually results in the upsurge in viscosity.

### 3.12 Heat stability

The heat stability of plant-derived milk varies depending on the specific plant source and processing techniques. Plant-based milks typically exhibit varying heat stability compared to cow's milk, owing to their varied compositions. The increased fat content of coconut milk allows it to survive higher temperatures without curdling. Coconut milk is documented to have superior heat resistance compared to numerous other plant-derived alternatives. On the other hand, almond milk can occasionally separate or curdle when heated. This discrepancy may become more apparent when exposed to higher temperatures or when combined with hot beverages such as coffee. When incorporating plant-based milk analogues into cooking or heating processes, it is crucial to consider the distinct attributes of each variety. Heating may cause some separation or changes in texture, though this does not necessarily imply spoilage or safety concerns. Soy milk exhibits superior thermal stability in comparison to other plant-based alternatives. It can be heated without substantial separation or curdling, making it suitable for cooking and baking.

Cold plasma treatment can appreciably affect the protein structure in plant-based milk, reducing heat stability by disrupting the protein's secondary and tertiary assemblies through interactions with reactive species, leading to protein unfolding, aggregation, and rupture of peptide bonds, which eventually impact the milk's ability to tolerate high temperatures without curdling. Analysing cold-plasma-treated plant-based milk analogues using DSC can help understand how this treatment affects the thermal properties and stability of the components present in these products. DSC, or differential scanning calorimetry, aids in determining thermal stability. The loss in thermal stability is due to the loosening of peptide bonds and to surface etching (Basak & Annapure, 2022). Dabade et al. (2023) examined the thermal stability of soy protein isolate. They stated that the control sample required a denaturation temperature of 215.05 ± 0.30, which was higher than the denaturation temperature required for samples treated with cold plasma, which dropped the temperature to 209.05 ± 0.66, indicating that ACP had denatured the molecule's fractional structure. Ji et al. (2018) reported similar results in their investigation of the impact of CP therapy on peanut protein. The results showed that the denaturation temperature dropped from 85.07 to 82.53 °C as the treatment period increased, indicating a decrease in the treated sample's thermal stability relative to the untreated control. According to the results, there was less protein isolate aggregation due to weak intermolecular forces, leading to weaker peptide interactions. One major restriction on CP modification is the reduction in the heat content for aggregation and denaturation. However, in their study, J. jiao Yu, Huang, et al. (2020) used CP to chemically alter peanut protein in the presence of lactose, which corrected the problem. According to the study, the sugar may have contributed to maintaining the rigidity and integrity of the protein structure, which, in turn, may have helped raise the temperature and enthalpy of denaturation of the protein. However, it was a sugar-specific effect, and thermostability decreased significantly when similar research was conducted in the presence of sesbania gum (Yu, Ji, et al., 2020). The disruption of intramolecular forces in proteins by CP may decrease their thermostability, potentially limiting their use in the food chain. In a study conducted by Ji et al. (2020), the impact of ACP on the denaturation enthalpy and denaturation temperature of dextran and peanut protein isolate (PPI-Dex) was investigated. They found that as ACP treatment time increased from 0 to 1.5 min, PPI's thermal stability and intermolecular interaction (denaturation temperature) decreased. This was likely due to the possibility that PPI was covalently bound to dextran and in an unfolded state. Similar results were observed for denaturation enthalpy, which decreased as the PPI-Dex conjugates' hydrophilic and hydrophobic interactions strengthened. This phenomenon is related to glycosylation, an exothermic reaction that also decreases the denaturation enthalpy.

### 3.13 Particle size distribution

The size of a beverage constituent particle affects both its mouthfeel and sedimentation stability over time. The active, strong plasma species, along with free radicals, disrupt the protein's secondary and tertiary structures, which are central to changes in the treated material's shape due to their surface-etching activities. The Uniform particle size is intended to be produced by short-term plasma treatment (Mahdavian Mehr & Koocheki, 2020). In their research, Chen et al. (2024) found that the ACP treatment decreased the size of coconut milk droplets, especially at 60 kV for 60 s (from 28.0 μm to 18.6 μm). While excessive ACP treatment led to droplets clustering and growing in size, moderate ACP treatment decreased droplet size. The droplet size was mostly unaffected by insufficient ACP treatment, resembling that of untreated coconut milk. Chen et al. (2023) similarly demonstrated that adjusting ACP treatment conditions can alter the structure of coconut protein. Eazhumalai et al. (2022) observed no appreciable alteration in the mean particle size of the oat milk particles following plasma treatment. However, a notable difference in the particle-size distribution of the constituents was observed. The largest particle aggregate, measuring 22.962 mm, and the smallest particles, measuring 0.279 mm, were found in oat milk treated at the maximum plasma input voltage of 230 V. Yu, Huang, et al. (2020) in a study on flaxseed protein reported that during the first 60 s of the APPJ treatment, there was a small increase in the particle size. On the other hand, the protein aggregated into large particles when the APPJ treatment period was increased to 90 s. The reason for this is that protein molecules exhibit reduced electrostatic repulsion in the vicinity of the isoelectric point. Ji et al. (2018) observed comparable outcomes, demonstrating a substantial increase in the mean particle size of peanut protein isolate (PPI) micelles treated with CP from 996 ± 28.0 to 1208 ± 33.2 nm compared with native PPI micelles. Nevertheless, the mean particle size of PPI micelles decreased from 1112.7 ± 7.2 to 1062.3 ± 29.4 nm after 1–3 min of CP treatment, then increased again to 1208 ± 33.2 nm after 4 min of CP treatment. The PPI solutions subjected to intense ion bombardment (electrons, ions, and other active species) may have led to the discovery of active sites, as this increased the particle size of PPI micelles after the CP treatment. Additionally, it could strengthen hydrophilic interactions and the bond between protein molecules and water micelles. The 3-min CP treatment may have caused protein micelles to aggregate, thereby significantly increasing the mean particle size of PPI micelles. In the case of soy protein isolate (SPI), Zhang et al. (2021) similarly found a similar outcome: processing for 1 to 2 min at 80 Hz slightly decreased the SPI particle size, but as the ACP duration increased, the protein particles began to clump together and caused the particle size to grow.

### 3.14 Scanning electron microscopy (SEM)

The structural analysis of plant-based milks is performed using an optical microscope and scanning electron microscopy (SEM), which permit precise observations at the molecular level to evaluate the structure. Cold plasma is reported to cause mechanical shock due to its high energy, leading to molecular disruption (X. Wu et al., 2021). Chen et al. (2024), in their study on ACP-treated coconut milk, stated that ACP treatment led to a large-droplet conversion to small ones, particularly at 60 kV for 60s, compared to the large, separated, or non-flocculated ones present in the control sample. The formation of smaller droplets results in a lower friction coefficient, which in turn yields a creamier milk taste. Eazhumalai et al. (2022) observed a similar outcome and stated that the plasma-treated oat milk contained small milk constituents in an aggregated manner, owing to intramolecular particle binding resulting from surface modification. Dabade et al. (2023) studied the surface structure of Soy Protein isolate (SPI) treated with ACP and found that the treated SPI surface was smooth, porous, and homogeneous, with aggregation, whereas the control sample was rough and heterogeneous. Yu, Huang, et al. (2020) in their study on flaxseed protein specified sequential variations in flaxseed protein microstructure persuaded by sustainable plasma active species as a result of APPJ treatment, viz., flaxseed protein denaturation, flaxseed mucilage polysaccharide adornment on insoluble protein particles and mild cleavage of peptide chains. (Yu, Ji, et al., 2020) in their study on lactose conjugate and high-temperature peanut protein (L-HPPI) conjugate specified that the surface structure of L-HPPI conjugates turned out to be less uniform and compact than that of the non-grafted HPPI.

### 3.15 Attenuated Total reflection Fourier transform infrared (ATR-FTIR) spectroscopy

To detect changes in functional groups upon plasma treatment, FTIR analysis is widely employed. The secondary structure of the protein can be modified using the CP treatment, as characterized by its voltage, highly charged gases, and duration of exposure. According to Toldrá et al. (2020), CP releases reactive oxygen and nitrogen species (RONS), which induce changes in secondary structures, disrupt sheets, and alter amino acid side chains, thereby affecting protein structure. Anbarasan et al. (2023), in a study on soy milk using plasma bubbling (PB), observed changes in the peak intensities of the amide group, suggestive of modified protein secondary structures. With the increase in treatment time from 5 to 25 min at 100 V, a reduction in the α-helix fraction (26.7% to 19.7%) and an increase in the β-sheet fraction (42.3% to 52.0%) were observed. A corresponding effect was observed at 200 V, causing a reduction in α-helix fraction (27.7–21.1%) and an increase in the β-sheets (36.8% to 46.90%), indicative of the PB-induced secondary structural changes in soymilk proteins. Additionally, unlike at 100 V, the turn fractions remained present (turns: 3.3%) in the 200 V bubbling treatment even after 25 min, which is attributed to differences in the nature of reactive species generated at different voltage levels. In a study on soy protein isolate (SPI), Dabade et al. (2023) observed that the β-sheet content in treated samples increased from 47.04% to 75.78% as treatment time and intensity were extended. Additionally, a reduction in the vibrational intensities of N—H amine bonds, X–H triple bonds, and X–H double bonds was observed, leading to partial breakdown of peptide bonds and changes in the triple bond region of proteins. A shift in the ratio of β-turns and β-sheets, as well as alterations in intermolecular β-sheet antiparallel structures, were also observed, along with a modest decrease in FTIR transmittance. In another study on peanut protein isolate and dextran (PPI-Dex) conjugates by Ji et al. (2020), it was observed that after 0.5 min of ACP treatment, there was an increase in hydrophobic β-sheets and a decrease in hydrophilic β-turns compared with the untreated sample. With an increase in ACP treatment time, a decrease in the content of β-sheets, random coils, and α-helices was seen, while the content of β-turns increased. The reduction in α-helices, attributed to covalent connections between polysaccharides and the ε-amino group in the α-helix region, led to PPI unfolding. Additionally, a decrease in β-sheets and an increase in β-turns indicated improved surface hydrophilicity of PPI. Ji et al. (2019) investigated the impact of ACP on peanut protein isolate powders, finding that the β-turn content increased after three minutes of treatment. In contrast, the β-sheet content decreased from 32.34% to 24.00%. The observed changes suggested improved surface polarity and hydration of PPI. In a separate study by Ji et al. (2018) on peanut protein isolate (PPI), the CP-treated sample exhibited a distinct secondary structure compared to the untreated sample. The content of random coils and β-sheets significantly increased during the three-minute CP treatment. This increase in random coil content enhanced adsorption at the oil-water interface, enhanced emulsifying properties, and partially denatured the protein structure. After four minutes of CP treatment, the α-helix content decreased from 14.07% to 9.97%. Similar changes were noted in the β-turn content. This shift indicated a transition from a tight, ordered structure to a loose, disordered one under high-energy ion bombardment.

### 3.16 SDS-PAGE analysis

SDS-PAGE (Sodium Dodecyl Sulfate Polyacrylamide Gel Electrophoresis) is an important approach in protein analysis, providing valuable insights into the composition and characteristics of protein samples, including those found in plant-based milk analogue products. SDS-PAGE analysis facilitates the determination of protein molecular weight, evaluation of protein sample purity and homogeneity, examination of protein complexes or numerous subunits within a sample, and confirmation of protein expression or identification of protein degradation products. It is believed that, following plasma therapy, band intensity will decrease due to protein crosslinking and its structural changes, viz., unfolding, aggregation, and fragmentation. Anbarasan et al. (2023), in a study on soy milk, reported that in plasma-treated samples, no new protein band was observed in the SDS profile. However, using voltages (100 and 200 V) and treatment time of 25 min, a substantial diminution in the intensity of β-subunit (48–63 kDa) of β-conglycinin was observed. In addition to this reduction in glycinin-A and glycinin-B subunits at 100 and 200 V treatments after 25 min, an evocative change was observed, suggestive of the changes caused by PB, attributed to protein−protein cross-linking and the formation of small protein fragments. Dabade et al. (2023) noted that the primary bands discernible on the SDS-PAGE of the treated SPI samples were glycinin (A: 29–33 kDa) and β-conglycinin (α’: 68–72 kDa, α:63–66 kDa, and β: 44–51 kDa). As the ACP treatment intensified, a β-conglycinin (∼71 kDa) fadeout area was observed, which eventually led to a decrease in protein allergenicity. There was a noticeable drop in the molecular weight bands between 71 kD and 51 kD. The work by Zhang et al. (2021) reports that, after plasma treatment, the molecular weight of the bands already present either changed noticeably or new bands containing unknown proteins formed. These results implied that plasma exposure did not result in protein subunit degradation or agglomeration, nor in the formation of covalent bonds between proteins and other molecules. Venkataratnam et al. (2020) explored the effect of cold plasma on the protein solubility of the major peanut allergens (Ara h1 and Ara h2) using SDS-PAGE. Ara h1 and Ara h2 are distinct proteins found in peanuts (*Arachis hypogaea*) and are recognised as primary allergens that induce allergic reactions in individuals with peanut allergy. The results demonstrated that, with an increase in treatment time up to 15 min, there were no alterations in the intensity of the Ara h1 or Ara h2 allergens in whole peanuts (WP). Compared with the control, there were a few slight differences in intensity as the treatment progressed. The SDS-PAGE analysis of defatted peanut flour (DPF) showed a notable shift in band intensities compared to WP. It was found that the Ara h1 intensity decreased, and the band associated with the Ara h2 doublet became dim after a 60-min treatment session. Variations in the observed effects of plasma on WP and DPF could be attributed to alterations in the food matrix, surface characteristics, and the way that plasma interacts with other food components, such as carbohydrates and lipids. The study revealed that cold plasma was effective in reducing the solubility of peanut protein and altering the structure of allergens, thereby decreasing antigenicity. Meinlschmidt et al. (2016) stated that direct and remote CAPP treatments decreased protein solubility and concurrently formed insoluble aggregates, which is why the protein bands in SDS-PAGE disappeared. In the electrophoretic profile, only soluble proteins are evident. Ji et al. (2020) examined the SDS-PAGE patterns of dextran and peanut protein isolate and found that the ACP-treated samples showed a dark broad band at the bottom and a broad band at the top of the running gel (PPI-Dex). The upper band required the synthesis of high molecular weight PPI-Dex conjugates. The result further demonstrated that ACP medication decreased the time required for protein glycosylation. The dark, broad band at the bottom was found to be caused by tiny molecular-weight subunits that were blasted by high-energy particles. Ji et al. (2018) studied the effects of dielectric barrier discharge cold plasma treatment on the physicochemical and functional properties of peanut protein. This study demonstrated no apparent distinction in the electrophoresis patterns of peanut protein isolate between cold-plasma-treated and untreated samples. The findings indicate that the CP treatment did not alter the basic structures and did not form any covalent linkages between proteins and other molecules (such as proteins, carbohydrates, or water micelles). The CP treatment did not have a substantial impact on the S—S bonds or noncovalent interactions between peanut protein components.

## 4 Impact of cold plasma on physicochemical and nutritional properties of plant-based milk analogues and proteins

### 4.1 pH

pH is a critical aspect of food quality, influencing sensory characteristics, shelf life and nutritional properties. Many researchers have reported a drop in pH following plasma therapy. The disparity in pH values post-plasma treatment can be attributed to numerous factors, viz., the type of plasma source (gliding arc discharge or dielectric barrier discharge), voltage and power settings, distance between the solution surface and the plasma generation area, use of buffering matrices, and treatment volumes. Moreover, X. Yu, Huang, et al. (2020) found that the transfer of plasma-generated reactive species into the treatment medium, particularly in solutions with variable protein concentrations, substantially impacts pH. Chen et al. (2024) stated that coconut milk treatment with atmospheric cold plasma (ACP) led to a decrease in pH owing to the production of active chemicals generated by high-voltage electricity. An analogous finding was reported by Dharini et al. (2023), who stated that the sesame milk pH decreased as a result of improved ionization and altered protein structure in the milk after plasma treatment. Eazhumalai et al. (2022) reported that an increase in input voltage and treatment time resulted in a decrease in pH due to the increased reactivity of plasma-generated radicals, which is always associated with higher voltage and longer exposure times. The researchers reported that, in treated oat milk samples, when input voltage and treatment duration were increased, pH values dropped more sharply. In a comparable investigation led by Muhammad et al. (2019), a decrease in pH of TNM milk treated with cold plasma was observed with increasing plasma exposure. This validates that the length of plasma exposure is a major factor in determining milk pH, which is often associated with increased solubility of reactive species and hydrolysis of milk water after plasma treatment. X. Yu, Huang, et al. (2020) in their study investigated flaxseed and revealed that the flaxseed protein dispersion's pH value progressively dropped from 9.0 ± 0.21 to 3.45 ± 0.15 following 240 s of APPJ treatment. The reduction was attributed to the reaction of H2O2 with N2O, which produced the acids H_3_O+, HNO_2_, and HNO_3_. Ji et al. (2018) similarly observed a slight reduction in pH (7.0 vs. 6.8) in the peanut protein solution after 240 s of DBD cold plasma therapy. However, contrary results were reported by Anbarasan et al. (2023) in their investigation of soy milk using plasma-bubbling PB. pH elevation in milk was detected when exposed to 100 V and 200 V, with treatment times of more than 25 min and 15 min, respectively. This can be attributed to the augmented formation of hydroxide (OH) concentration resulting from reactive oxygen and nitrogen species generated by PB.

### 4.2 Titratable acidity

The total acid concentration in a liquid is measured by Titratable acidity (TA). It is centred on acid neutralisation using the base NaOH. It is an efficient parameter for investigating the impact of plasma treatment on the flavour of plant-based milk analogues. Eazhumalai et al. (2022) reported that the TA of the plasma-treated oat milk increased from 0.054% lactic acid in the control sample to 0.162% lactic acid (230 V – 15 min) in the treated oat milk sample. An analogous finding was reported by Muhammad et al. (2019) in tiger nut milk, where TA increased from 0.118% in the control sample to 0.131 ± 0.002% (30 V-12 min) in the DBD plasma-treated tiger nut milk sample. The increase in the TA of the milk is due to the development of acidic components, viz., nitric acid, nitrous acid, and hydrogen peroxide, formed as a result of reactions between plasma reactive species and water. Additionally, the increase is attributed to the production of the carboxylic group from amino acid degradation and to the oxidation of aldehydes by O3 and OH- generated by the plasma (Ozen & Singh, 2020; Sharma & Singh, 2022). Chen et al. (2024) also reported an increase in the TA of coconut milk at higher power and exposure time using ACP. However, at lower voltage power and exposure time, no significant change was reported. Dharini et al. (2023), however, reported contradictory results for TA and reported a decrease in TA of sesame milk from 0.168% in the control to 0.161%, 0.146%, and 0.155% in sesame milk treated with PB for 10, 20, and 30 min, respectively. The researchers stated that the possible formation and dissolution of OH and other free radicals might have affected TA, thereby resulting in a decrease in TA.

### 4.3 TSS

TSS refers to the concentration and composition of dissolved solids that affect the sensory and textural characteristics of the plant-based milk analogues. The cold plasma treatment demonstrated no significant impact on Total Soluble Solids (TSS). Eazhumalai et al. (2022), in their study on oat milk, reported that at an input voltage of 170 V, the TSS of oat milk increased. However, with the input voltage increased to 200 V and 230 V, the TSS increased substantially. Overall, an insignificant change in the TSS of the treated oat sample was found compared to the control sample, attributed to moisture loss from the treated sample (Ozen & Singh, 2020). The analogous finding was reported by Muhammad et al. (2019) and Chen et al. (2024) in tiger nut milk and coconut milk treated with DBD plasma and ACP, respectively, owing to the incapability of the plasma active species in reaching the macromolecules (starch, protein, and fibres) present in the food matrix.

### 4.4 Colour

Colour is one important sensory attribute. It is an essential component of quality that affects the product's suitability and, consequently, its success. The disintegration of lipid, protein, and carbohydrate molecules in the milk treated with plasma results in changes to its optical characteristics. Chen et al. (2024) investigated how plasma therapy affected colour changes in coconut milk and found no significant change (*p* > 0.05) in the L* value when ACP was used. Compared with untreated coconut milk, b* values decreased and a* values increased after ACP treatment. The greater overall colour change in the coconut milk was found to be caused by an increase in exposure duration and voltage. The protein content of coconut milk is associated with its opaque white colour, and the ACP treatment is believed to promote proteolysis but with distinct protein compositions. The reaction between the proteins present in coconuts and the active species generated by the ACP treatment was expected to cause the colour changes. Dharini et al. (2023) observed colour differences in sesame milk in their study. The results showed that the a* and b* values increased, while the L* value decreased as plasma application increased. The substantial variance seen in all results suggests that sesame milk could be oxidised by plasma. However, the overall colour change (ΔE) values showed very little variation. Furthermore, the sesame milk treated with plasma did not exhibit any appreciable colour change. According to the study by Eazhumalai et al. (2022) on oat milk, there were minor variations in the milk's lightness, redness/greenness, and yellowness after plasma treatment, resulting in significant (*p* < 0.05) changes in the total colour difference (ΔE) values. Nevertheless, the milk's appearance showed no obvious changes. However, with an increase in input voltage and treatment time, an increase in the ΔE value was observed. The researchers stated that the aggregated particle components identified in the oat milk samples that had received plasma treatment delayed and refracted the light entering the sample, decreasing its apparent brightness and changing its colour.

### 4.5 Protein and fat

The outcome of cold plasma treatment is usually a decrease in protein content in plant milk. Eazhumalai et al. (2022) reported a decrease in the protein content of the oat milk compared to the control sample. The decrease in protein content was more pronounced with increasing input voltage and exposure time. The protein reduction is attributed to the interaction of ROS and RNS with protein molecules, resulting in changes in protein conformation and structure. A comparable finding was reported by Muhammad et al. (2019) in tiger nut milk, where a drastic reduction in protein content was observed from 0.45 g/100 ml in the control sample to 0.16 g/100 ml in the sample treated with DBD plasma. However, Chen et al. (2024) and Anbarasan et al. (2023), in their studies on coconut and soy milk, respectively, reported no significant change in protein content using ACP. In plasma-treated plants, the fat content of the plant milk shows no significant alteration. The researchers Chen et al. (2024) and Muhammad et al. (2019) reported no significant variation in fat content in coconut and tiger nut milk treated with ACP and DBD plasma, respectively.

### 4.6 Composition of fatty acid and lipid peroxidation

According to the investigation into the impact of plasma treatment on fatty acid composition, the ratio of unsaturated to saturated fatty acids (UFAs/SFAs) decreased with increasing voltage or time. This might be an outcome of more free reactions occurring for prolonged periods or at higher voltages, leading to the production of unstable compounds, active constituents, and free radicals (Sarangapani et al., 2017). The effect of plasma treatment on the fatty acid content of coconut milk was observed by Chen et al. (2024). It was reported that ACP affected the fatty acid composition. While oleic and linoleic acid levels decreased, capric and lauric acid levels in coconut milk did not differ substantially. Additionally, the concentration of caproic and caprylic acids increased. Theoretically, ACP administration is thought to promote the hydrolysis of long-chain fatty acids (LCFAs), thereby increasing the concentration of medium-chain fatty acids (MCFAs). There was an interaction between the active species generated by the ACP therapy and the unsaturated fatty acids (UFAs). Furthermore, the impact of ACP therapy on fatty acids was connected to the degree of unsaturation. The amount of unsaturated fatty acids increased with the ease with which they could react with active species and break down double bonds. However, Alves Filho et al. (2019) observed an increase in fatty acids (oleic, linoleic, palmitic, and stearic) following plasma treatment in their cashew nut research.

Negative effects on sensorial attributes are mostly caused by oxidation of lipids, which ROS, RNS, and atomic oxygen usually bring on. The secondary oxidation byproducts produced by this process, including acids, ketones, alkanes, and aldehydes, further affect food's sensory attributes and result in lower sensory scores in plasma-treated food (Muhammad, Xiang, et al., 2018). Numerous studies indicate that mild ACP therapy is considered safe to take (Dzimitrowicz et al., 2021). TBA (Thiobarbituric Acid), TBARS (Thiobarbituric Acid Reactive Substances), and lipid hydroperoxide assays are commonly used to assess lipid oxidation levels in various food products, including plant-based milk analogues. Rancidity is usually evaluated using the peroxidation value (POV). Chen et al. (2024) examined the effects of ACP on POV and TBARS in coconut milk. Post ACP treatment, a slight shift in perception of coconut milk was observed, indicating that the milk did not taste awful. The TBARS value increased substantially after receiving ACP therapy, rising from 0.062 mg/kg to 0.088 mg/kg. After ACP treatment at 70 kV, there was an upsurge in both cases, attributed to the oxidation of unsaturated fatty acids in coconut milk, enabled by ROS and RNS. Nevertheless, the changes were undetectable because the coconut milk had very little lipid oxidation and little oil oxidation to affect sensory perception. In contrast, Dharini et al. (2023) conducted a TBARS analysis on sesame milk treated with plasma and found that the malondialdehyde concentration in the milk increased from 0.39 to 0.83 μg MDA/l with increasing plasma exposure time from 10 to 30 min. Muhammad et al. (2019), in their investigation of tiger nut milk (TNM), reported that plasma treatment dramatically elevated (*P* < 0.05) the quantity of TBARS in all treated samples compared with the control. The activity of ROS, such as atomic oxygen and OH radicals, which may have caused lipid oxidation, explains why the rise was more noticeable when the treatment duration was extended.

### 4.7 Amino acid profile analysis

The quantity and composition of amino acids affect nutritional value. It is believed that ACP causes hydroxylation, sulphonation, nitration, and dimerization in aromatic and basic amino acids containing sulfur. It is well known that the side chains of aromatic and sulfur-containing amino acids are especially susceptible to oxidation, especially those of cysteine residues. Chen et al. (2024) examined the effect of amino acid concentration in coconut milk following ACP therapy. The findings showed that as treatment length and voltage power increased, total amino acid content decreased. This decrease can be explained by the interaction between amino acids and ROS and RNS produced by the ACP therapy. After the ACP treatment, the Asp content of coconut milk did not change much, but its Glu level did somewhat rise, suggesting that the amino acids related to flavour were less sensitive to the ACP treatment. However, after the ACP treatment, especially at 70 kV for 90 s, the amino acid concentration, specifically Met and Cys, decreased. This occurred as a result of the ACP-formed ROS and RNS promoting the sulfoxylation of Met and the creation of disulfide bonds and sulfhydryl sulfonation in Cys. The Phe and Tyr content of coconut milk likewise dropped after the ACP treatment, indicating that the distance between the OH and benzene ring was a simple case of the hydroxylated and nitrated benzene ring of aromatic amino acids. Concurrently, there was a decrease in the levels of Arg and Lys, linked to the amide adduct formed between amino groups and reactive oxygen species. Consequently, it was easy to carry out the ACP-induced hydroxylation, sulphonate, nitration, and dimerization of the aromatic, basic, and S-containing amino acids. Dharini et al. (2023), in their study on sesame milk treated with plasma bubbling, reported that, amongst the other important amino acids, arginine was less abundant than aspartic acid and glutamic acid. The presence of amino acids leucine and arginine was reported to be in higher quantities in the plasma-treated sesame milk. Additionally, it was observed that plasma-treated sesame milk showed an increase in levels of free aspartic and glutamic acid. Plasma-treated sesame milk (30 min of bubbling) showed higher total free amino acids than the untreated sample. The study on soy protein isolate (SPI) reported that with an increase in the ACP time, a progressive decrease was observed in the -SH group (Zhang et al., 2021). Additionally, a very small effect was observed on -SH levels at various plasma frequencies. The authors stated that the sulfhydryl groups breakdown in SPI is probably due to oxidative conditions known to promote impairment of sulfur-containing amino acid residues, such as cysteine and methionine. These results demonstrated that plasma species oxidised SPI and suggested the formation of disulfide (-S-S-) crosslinks within or between protein polypeptide chains. According to Ji et al. (2020), the ACP treatment decreased the levels of lysine and phenylalanine in mixtures of peanut protein isolate and dextran (PPI-Dex), but it had no effect on arginine composition because it mainly altered the surface of the polymeric materials, hiding arginine within the PPI molecule. Ji et al. (2018) found that treating peanut protein (PP) with CP may alter amino acid side chains in various ways, including the –SH groups. The results show that throughout the three-minute ACP treatment, the total free-SH content decreased steadily and eventually reached approximately half its initial value due to oxidation of free-SH groups into disulfide bonds. Three minutes later, there was a little increase in the total free-SH concentration, which may have resulted from the high-energy ion bombardment breaking disulfide bonds. However, following two minutes of CP treatment, a significant increase in PPI's exposed free-SH content was observed, indicating the formation of unfolded molecular structures. After 2 min, the free-SH level exposed in the treated samples decreased due to the formation of protein micelles.

## 5 Impact of cold plasma on microbial inactivation and antigenicity of plant-based milk analogues and proteins

### 5.1 Microbial inactivation

The objective of the microbiological analysis of plant-based milk analogue samples subjected to plasma treatment is to assess the effect of the treatment on microbial load reduction. The efficacy of cold plasma in eliminating microorganisms in different types of plant-based milk equivalents depends on several factors, including treatment duration, plasma intensity, gas composition, and proximity to the plasma source. The effectiveness of cold plasma inactivation against different microorganisms is also influenced by the microbial species, the product's pH, and the bacteria's surrounding environment (Los et al., 2020). It is essential to optimise these parameters to achieve the desired level of microbial reduction while maintaining the quality of various plant-based milk analogues. Cold plasma is generated by applying an electrical field to a gas, such as air or nitrogen, at reduced pressure. This process produces extremely reactive species, such as ions, electrons, radicals, and UV radiation, which interact to deactivate microorganisms. Cold plasma can inactivate microorganisms through several mechanisms, including disruption of cell membranes, DNA damage, protein denaturation, and cell lysis. These combined actions result in the decrease or eradication of microbial populations when applied to plant-based milk analogues. The oxidation of cellular DNA and lipid peroxidation result from the susceptibility of unsaturated fatty acids to attack by OH radicals and from protein denaturation caused by the oxidation of amino acids. It is proposed that the penetrating bombing of radical's primes the cell wall's superficial lacerations, which ultimately results in its death. This process is termed etching, and it depends principally on reactions amongst the charged plasma and the substrate molecules. The charged plasma is said to accumulate on the outer surface of the cell membrane of Gram-negative bacteria, resulting in an electrostatic force that ultimately primes the cell membrane for rupture. However, Gram-positive bacteria are not reported to exhibit any such observable morphological changes. Additionally, in many cases, the microbial cells are made nonviable or killed by the reactions amongst the inner cell biomaterials and plasma generated reactive species that can diffuse through the cell membrane (Mao et al., 2021).

According to Dharini et al. (2023), plasma bubbling (PB) exposure duration was found to significantly reduce the microbial burden in sesame milk. The initial bacterial and coliform loads in the raw sesame milk were 7.61 log CFU/ml and 6.92 log CFU/ml, respectively. As plasma bubbling time increased, the microbial load decreased. The total bacterial and coliform load was found to be reduced by 0.21, 0.34, and 0.64 log drop and 0.66, 1.15, and 1.82 log reduction, respectively, at 10, 20, and 30 min, with the assistance of the PB. This suggests that using PB decreased the bacterial load in sesame milk treated with plasma. The reduction is attributed to the higher concentration of reactive species in milk, which reduces the microbial load. These dissolved reactive species may have been the catalysts for the synthesis of long-lived antibacterial species, such as hydrogen peroxide and the peroxyl radical. Plasma bubbling has also been demonstrated to greatly increase the solubility of reactive species in addition to plasma therapy. In a separate study, Eazhumalai et al. (2022) reported that processing oat milk using plasma at 230 V for 15 min decreased the microbial loads of mould, yeast, and bacteria by 1.57, 2.18, and 2.18 log CFU/ml and 2.18 log, respectively. However, the least amount of bacteria, yeast, or mould was reduced when plasma therapy was used for five minutes at 170 V. The reductions were 0.29 and 0.87 log CFU/ml, respectively. These findings highlight the important roles of longer exposure times and higher input voltages in the decline in log counts. Muhammad et al. (2019) studied the application of DBD plasma to inactivate bacteria in tiger nut milk (TNM). Microorganism counts decreased with increasing plasma treatment duration. Initial counts of bacteria, mould, and yeast for the untreated TNM were 4.47, 4.27, and 4.47 log CFU/ml, respectively. These numbers were significantly lower after 2 to 12 min of plasma therapy. In particular, the counts of bacteria, mould, and yeast dropped by 0.17 and 0.2 log CFU/ml in 2 min, and by 0.37 and 0.34 log CFU/ml in 4 min. Moreover, the counts of bacteria, mould, and yeast dropped by 3.6 and 3.5 log CFU/ml in 6 min, and by 3.5 and 3.3 log CFU/ml in 8 min. In compliance with the national sanitary standard for fruit and vegetable beverages (GB19297–2003), which states that the total bacterial count should be ≤100 CFU/ml and the counts of moulds and yeasts should be ≤20 CFU/ml, the microflora counts were below the detection limit (< 1.0 log CFU/ml) after 12 min of plasma treatment. The data showed a pattern of steady inactivation from 2 to 8 min, followed by a dramatic drop to undetectable levels after 12 min of plasma treatment. The rapid deactivation at low pH in the advanced stage could be due to the production of plasma-reactive species, as the pH fell from 7.03 to 5.4 after a 12-min treatment. This abrupt decline in log counts may possibly be linked to an increase in the production of peroxynitrite and hydroperoxyl radicals, which can damage bacterial cell membranes and have synergistic effects. Thus, the studies concluded that cold plasma effectively helps accomplish microbial inactivation in plant-based milk analogues.

### 5.2 Antigenicity analysis (by ELISA)

Allergenicity is recognised by allergen-specific immune cells, which prime for specific immunological reactions. They consist of immunoglobulin E (IgE)-mediated or non-IgE-mediated. IgE is the exclusive immunoglobulin that facilitates a prompt allergic response. The plant-based alternatives, viz., nut milk alternatives and soymilk, can be a substantial concern for customers, as they are the cradles of food allergens, thereby posing allergenicity. The use of thermal treatments, viz., boiling, steaming, and autoclaving, can possibly help reduce the IgE-binding capacity of most allergens, which is a principal reason for seeds being heat-treated before milk alternative extraction. Dhakal et al. (2014) assessed the effects of non-thermal high hydrostatic pressure (HHP) on almond milk and reported a decrease in the relative immunoreactivity of almond major proteins. Another non-thermal technology that has been recognised to be effective in reducing antigenicity is cold plasma. The technique for determining antigenicity is ELISA. Numerous dietary allergies have been demonstrated to have their antigenicity reduced by either direct or remote cold plasma. The process by which allergens are reduced involves interactions between proteins and reactive species that are created during plasma processing. These species can cleave peptide bonds and alter protein conformation; on the other hand, proteins can be crosslinked, denatured, or oxidised, which decreases their solubility by forming aggregates (Chizoba Ekezie et al., 2018). Anbarasan et al. (2023) studied the effect of plasma bubbling (PB) on Soybean Kunitz trypsin inhibitor (SKTI)- a protein compound found in soybean milk which can induce allergic reactions in the human body. At 100 and 200 V, the antigenicity was reduced to 60.95 ± 8.03% and 38.99 ± 17.7%, respectively, after 25 min of exposure time. This reduction in antigenicity is attributed to the alteration in the protein fraction. Zhang et al. (2021) used sera from five individuals with soybean allergy to examine the soy protein isolate (SPI) ‘s ability to bind IgE using iELISA. The most effective group in removing allergens was the one that underwent a 5-min 120 Hz treatment; this group's total SPI bound to IgE was reduced by up to 75%. In contrast, the group treated with samples induced at 100 Hz showed a slight alteration over 1 to 5 min, which then decreased slightly at 10 min. As the ACP time increased, the sample group treated at 80 and 120 Hz had a gradual decrease in residual allergenicity within 5 min, followed by a notable increase at 10 min. Venkataratnam et al. (2020) investigated the antigenicity of defatted peanut flour (DPF) and whole peanuts (WP) for Ara h1 and Ara h2 using competitive ELISA. They discovered that Ara h1’s antigenicity decreased with increasing plasma doses of both WP and DPF. By comparison, Ara h2 in WP showed reductions in antigenicity of 18% to 46% in 15 to 60 min, respectively. DPF demonstrated a greater reduction in antigenicity than WP, with reductions of 31% to 42% at 15 and 30 min, respectively. Moreover, differences in how some allergens lose their antigenicity are due to changes in their protein structures. However, Alves Filho et al. (2019) reported a contradictory result, suggesting that plasma did not affect the nuts' allergenicity. He conducted a direct ELISA using processed cashew nut extracts and a rabbit anti-cashew antibody, reporting very minor differences in binding by the rabbit polyclonal antibody. Human IgE binding to the processed cashew nut extracts demonstrated very slight differences, which is consistent with this. The statistically nonsignificant change in binding of the human cashew allergy or the rabbit anti-cashew IgE to the processed cashew nut extract samples thus indicates that there was no detectable influence of plasma on the allergenicity of the cashew nuts. Meinlschmidt et al. (2016) examined the effect of cold atmospheric pressure plasma on the immunoreactivity of soy protein isolate by sandwiching ELISA using three mouse anti-Gly m5 mAbs (Izimab-Glym5–3, Izimab-Glym5–4, and Izimab-Glym5–5). Applying pure CAPP significantly increased soy immunoreactivity to up to 100%, according to the data. The efficacy of remote CAPP treatment was demonstrated by the immunoreactivity being reduced by up to 89% after 90 min of therapy. Thus, it may be concluded that both direct and distant CAPP efficiently reduce soy Gly m5 immunoreactivity. Overall, the researchers concluded that ACP can be used for reducing allergenicity in plant-based milk analogues.

## 6 Conclusion

Plant-based diets are increasingly recognised as people prioritise function, sustainability, and well-being. Plant-based milks can help address the United Nations Sustainable Development Goals (SDGs) related to carbon emissions. Nutrient-enhanced plant-based milk analogues are a better option for people with lactose intolerance or milk protein allergies. The market for plant-based milk analogues is growing. Since more people are consuming plant-based milk analogues than traditional animal milk products, the industry should focus on advancing their sustainability, as well as their nutritional, sensory, physical, and chemical properties. As a result, cold plasma serves as a potential non-thermal processing technology for plant-based milk analogues, demonstrating positive results in microbial decontamination while maintaining nutritional and sensorial quality. Additionally, it has the potential to modify the structure and composition of proteins using active plasma species, which in turn results in alterations in the functional properties of plant milk. The commercial scaling up of plasma-treated milk analogues, however, remains a concern. The loss of amino acids during plasma treatment may result in proteins that are less nutritionally appealing. Therefore, further exploration is required to assess the nutritional value of these proteins that have undergone CP treatment. Long-term or high-voltage treatments have been found to be detrimental to most functioning aspects. This means that, to maximise the process parameters and extract the desired functionality from the protein, a well-considered technique is needed. CP effectively improved the gelation properties of plant proteins; however, extended treatment times can fragment the proteins into smaller pieces, reducing their ability to bind water. In addition, the industry has been hindered by the lack of a legislative framework for the cold plasma treatment of food systems. The acceptable limits for human exposure to and usage of CP should be investigated as soon as possible to facilitate industry implementation. The literature provides little information on the toxicological evaluation of these entities' effects, particularly those of reactive species (UV, ozone). It is necessary to analyse the feasibility of producing additional secondary products.

## Ethics approval consent

Not applicable.

## CRediT authorship contribution statement

**Entesar Hanan:** Writing – review & editing, Writing – original draft, Visualization, Validation, Software, Resources, Formal analysis. **Shivangi Srivastava:** Writing – review & editing, Visualization, Validation, Resources, Formal analysis, Data curation. **Aamir Hussain Dar:** Writing – review & editing, Visualization, Validation, Supervision, Investigation, Funding acquisition, Formal analysis, Data curation. **Kshirod Kumar Dash:** Writing – review & editing, Writing – original draft, Visualization, Validation, Supervision, Software, Resources, Project administration, Methodology, Investigation, Funding acquisition, Formal analysis, Data curation, Conceptualization. **Vinay Kumar Pandey:** Validation. **Rafeeya Shams:** Writing – review & editing, Visualization, Validation, Resources, Investigation, Formal analysis. **Sharath Kumar:** Writing – review & editing, Visualization, Validation, Software, Resources, Funding acquisition, Data curation. **Ufaq Fayaz:** Writing – review & editing, Validation, Supervision, Resources, Investigation, Funding acquisition, Data curation. **Ayaz Mukarram Shaikh:** Writing – review & editing, Visualization, Validation, Software, Data curation. **Kovács Béla:** Writing – review & editing, Validation, Software, Resources, Investigation, Formal analysis, Data curation.

## Consent to participate

Informed consent was obtained from all individual participants included in the study.

## Funding

Funding Project No. TKP2021-NKTA-32 has been implemented with support from the National Research, Development and Innovation Fund of Hungary, financed under the TKP2021-NKTA funding scheme.

## Declaration of competing interest

The authors declare that they have no known competing financial interests or personal relationships that could have appeared to influence the work reported in this paper.

## Data Availability

The datasets generated and analyzed during this study are available from the corresponding author on reasonable request.
